# Supplementary figures and images for: Characterization and genomic analysis of the Lyme disease spirochete bacteriophage ϕBB-1
Source: PLoS Pathog. 2024 Apr 1;20(4):e1012122. doi: 10.1371/journal.ppat.1012122 (PMC11008901; doi:10.1371/journal.ppat.1012122)

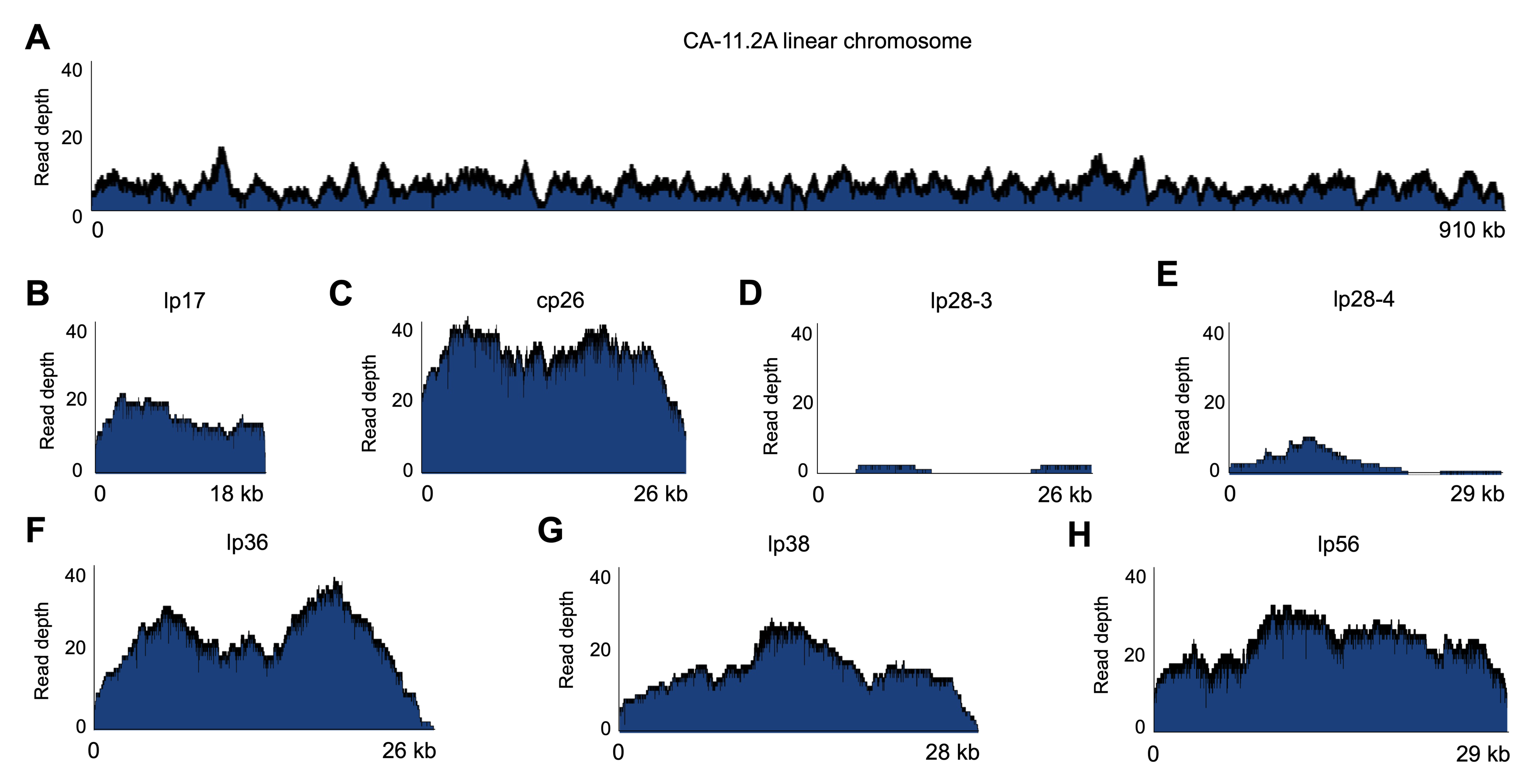

Supplement: S1 Fig — De novo assembly of packaged reads >5kb produced the indicated contigs. Read coverage was then mapped to each contig. (A–H) Read coverage across the CA-11.2A chromosome or indicated plasmids are shown. Coverage maps for the cp32s and lp54 are shown in Figs 6 and 9, respectively. (TIFF) [file ppat.1012122.s001.tiff]

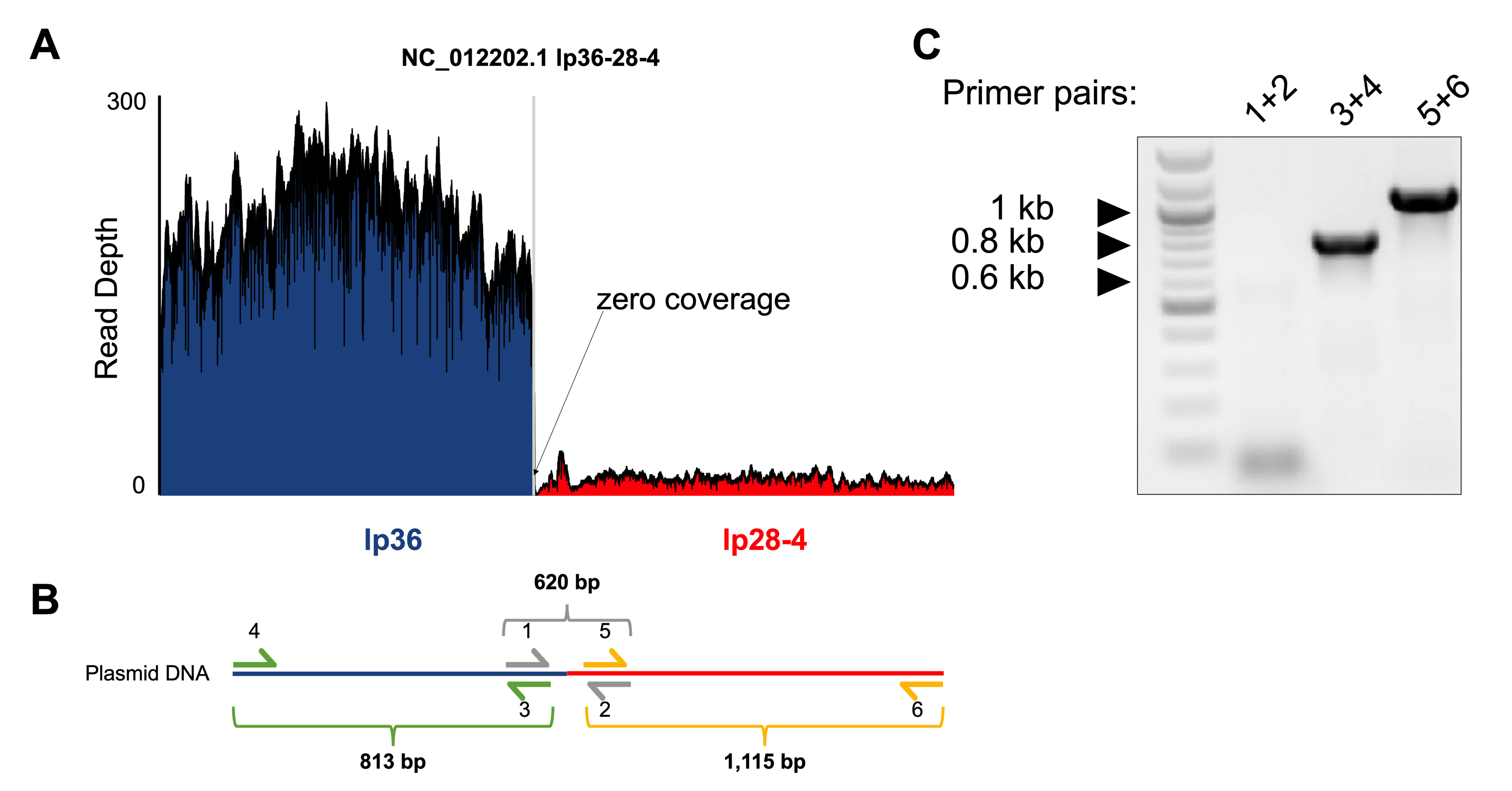

Supplement: S2 Fig — (A) The CA-11.2A genome was sequenced using long-read technology. Reads were aligned to the lp36/lp28-4 reference sequence (NC_012202.1) and read depth plotted. (B) Schematic of PCR design. Primers 1 and 2 flank the lp36/lp28-4 junction, with primer 1 annealing to lp36 and primer 2 annealing to lp28-4, creating a 620 bp product if joined. Primers 3 and 4 anneal to lp36 DNA, creating an 813 bp product if present. Primers 5 and 6 anneal to lp28-4 DNA, creating a 1,115-bp product if present. (C) The presence or absence of lp36, lp28-4, or lp36/lp28-4 was confirmed by PCR. (TIFF) [file ppat.1012122.s002.tiff]

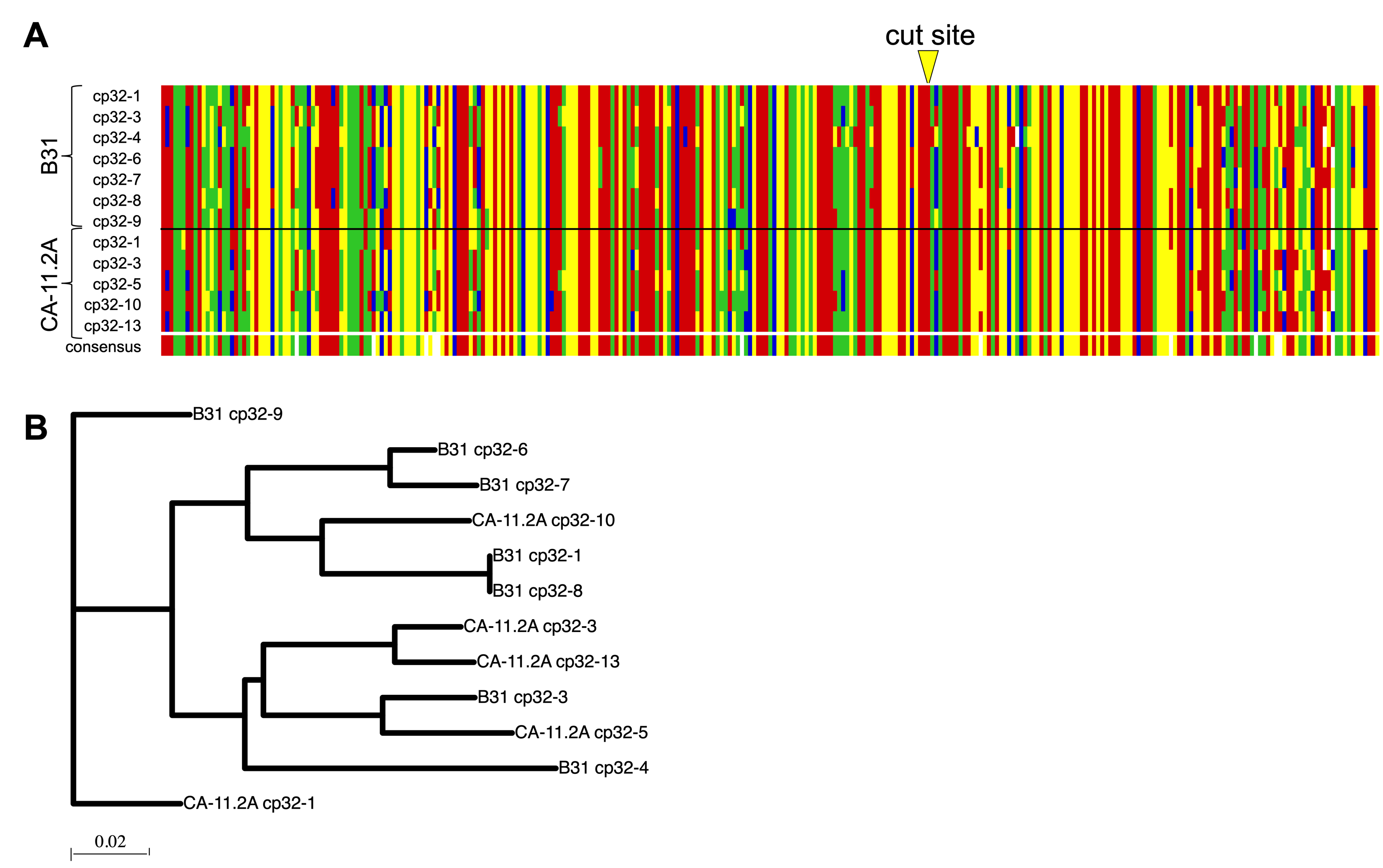

Supplement: S3 Fig — (A) The pac region from the cp32s in B. burgdorferi B31 and CA-11.2A were aligned by ClustalW. The cut site is indicated by yellow arrows. (B) Phylogenetic analysis of the pac regions was performed by constructing an uncorrected neighbor joining tree. (TIFF) [file ppat.1012122.s003.tiff]

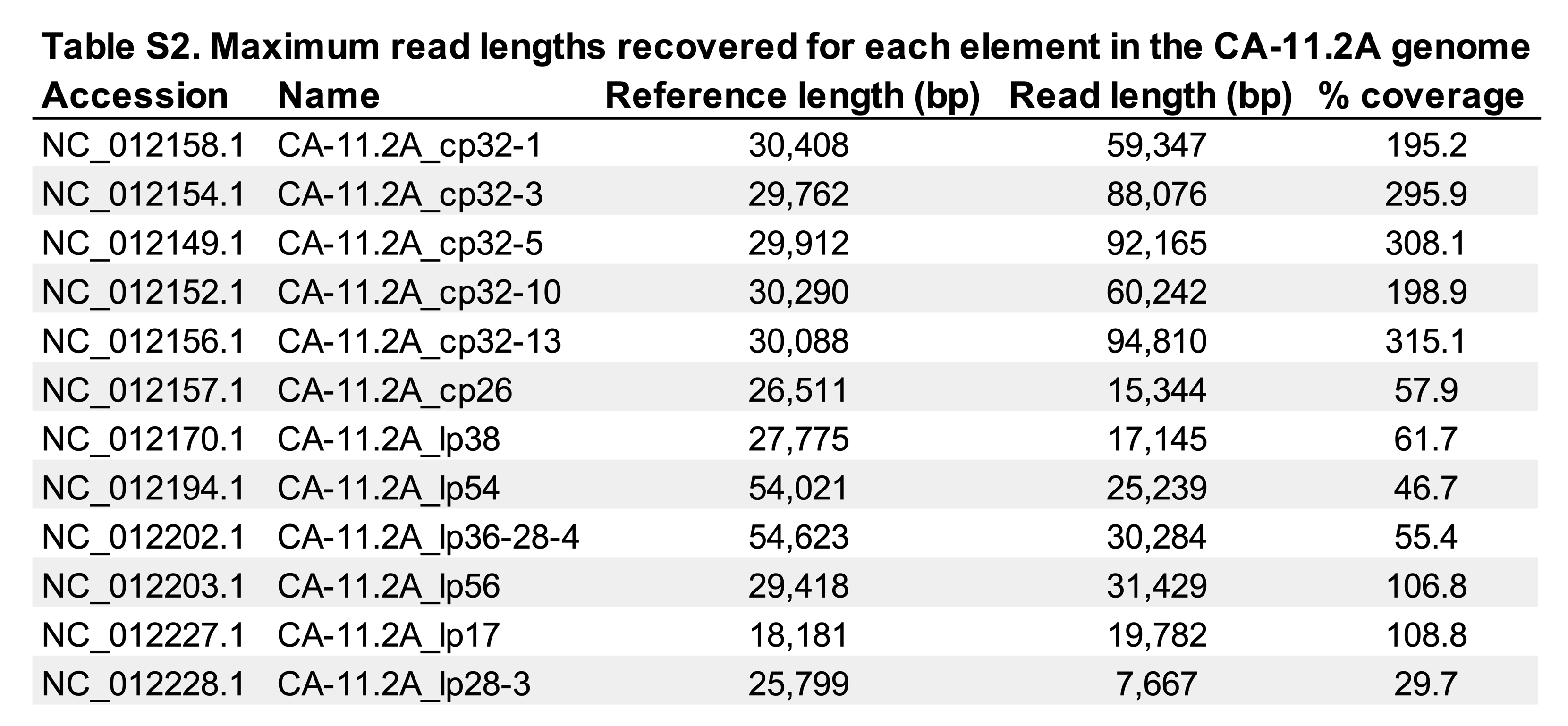

Supplement: S2 Table — (TIFF) [file ppat.1012122.s005.tiff]
