# Supplementary material for: Characterization and genomic analysis of the Lyme disease spirochete bacteriophage ϕBB-1
Source: PLoS Pathog. 2024 Apr 1;20(4):e1012122. doi: 10.1371/journal.ppat.1012122 (PMC11008901; doi:10.1371/journal.ppat.1012122)
Supplement: Attachment — Submitted filename: Response to Reviewers_submitted.docx [file ppat.1012122.s008.docx]

**Dear Editor,**

**Thank you for handling the review of this manuscript. We genuinely appreciate the feedback, and we think our response to the reviewers’ suggestions has improved the work.**

**We are grateful to the three Reviewers for their kind words and thoughtful summaries of our work.**

**The following is a point-by-point response to each concern. We hope the revised manuscript makes a scientific contribution consistent with the reputation of *PLOS Pathogens*.**

**The line numbers indicated below refer to those in the clean version of the manuscript.**

**Part II – Major Issues: Key Experiments Required for Acceptance**

**Reviewer #1:**

1. The authors claim that ϕBB-1 is a generalized transducing phage, but this seems based solely on finding low coverage reads of the linear chromosome with no obvious pac sites. Because generalized transduction wasn’t shown in this study, more evidence is needed to substantiate this claim. What was the size distribution of chromosomal reads? If there were a peak read length matching the headful packaging size of ϕBB-1, then this would be good evidence of generalized transduction.

**Thank you for the comment. As the reviewer notes, generalized transduction is the process by which phages can package any bacterial DNA (chromosomal or plasmid) and transfer it to another bacterium. Prior work demonstrates that ϕBB-1 is a generalized transducing phage as it can package shuttle vectors (which do not contain the *pac* region) and transfer them to other *B. burgdorferi* (PMID: 27811049). Our data are consistent and indicate that ϕBB-1 packages non-cp32 plasmids and fragments of the chromosome, supporting the conclusion that ϕBB-1 is a generalized transducing phage.**

**The average length for packaged chromosome reads is 7,858 bp, which is similar to the ~8,000 bp length of other low frequency plasmids packaged by ϕBB-1 (Fig 5D). This may indicate a packaging bias towards shorter DNA molecules for non-cp32 DNA molecules, which we now discuss on lines 161-64.**

2. It should be mentioned what the receptor for ϕBB-1 is. Is it known? And, if so, is it highly conserved across the genospecies?

**Unfortunately, the identity of the receptor(s) used by ϕBB-1 are not yet known. We now mention this on lines 296-298.**

3. Can you get viable ϕBB-1 in an lp54 minus strain, or is Ip54 essential?

**Lp54 is a highly conserved component of the *B. burgdorferi* genome that encodes OspA and OspB and is essential for *B. burgdorferi* to complete its enzootic cycle (see for example PMID: 10672174). To our knowledge, no one has successfully generated a lp54 null strain.**

**Reviewer #2: (No Response)**

**Reviewer #3: None noted.**

**Part III – Minor Issues: Editorial and Data Presentation Modifications**
Please use this section for editorial suggestions as well as relatively minor modifications of existing data that would enhance clarity.

**Reviewer #1:**

1. Figures 2A and 7B: The y-axis scales are not linear, and there is no way to interpret the data near the bottom. For example, the Figure 2A y-axis goes from 0 to 1 x 108, then to 2 x 108, etc., so there is no way to know what the copy number for B31 is. Replace the zeros with the correct numbers. The other graphs in the manuscript did it right.

**Thank you for pointing this out. The y-axes in Figures 2A and 7B have been modified to log10 scale and are shown below.**

**Figure 2.** ɸ**BB-1 phage titer, virion morphology, and proteomic analysis.** **(A)** Packaged, DNase-protected cp32 copy numbers in bacterial supernatants were measured by qPCR. Data are the SE of the mean of three experiments, ****p* <0.001. **(B)** Virions were purified from 4-L cultures of *B. burgdorferi* CA-11.2A and imaged by transmission electron microscopy. Representative images from two independent preparations are shown. **(C and D)** HPLC-MS/MS-based proteomics was used to identify proteins in two purified virion preparations. The SE of the mean of spectral counts for peptides associated with the indicated phage structural proteins are shown for each replicate. See also **Table S1** for the complete proteomics dataset.

**Figure 7. Shuttle vectors containing the cp32 *pac* region are preferentially packaged into ϕBB-1 virions. (A)** Schematic depicting the headful genome packaging mechanism. **(B)** After ethanol induction, ϕBB-1 virions were collected from CA-11.2A cells not carrying plasmid pBSV2 (No vector), cells transformed with empty pBSV2, or cells transformed with pBSV2 with the cp32-3 *pac* site (see Fig 6G for the cloned *pac* region). Copy numbers of pBSV2 packaged into ϕBB-1 virions were measured by qPCR. Data are the SE of the mean of three experiments, ****p*<0.001.

2. Figure 8B does not scale well, so some tiny nucleotides in the Logo are unreadable. A higher-resolution image is needed.

**A higher resolution TIFF image has been provided as a revised Figure.**

**Reviewer #2:**

1. Please provide additional information regarding the statistical analysis of the proteomic data provided in Figure 2. Overall, the capsid proteins were detected at low abundance. In contrast, OspC, OspA and GroEL were detected at high abundance. A greater discussion of how these data were analyzed is warranted.

**More detail on the statistical analysis of the proteomic data is included in the Methods section, Lines 452-55.**

**“Proteins were identified and quantified using Mascot [92] with a parent ion tolerance of 2.5 ppm and a fragment ion tolerance of 0.5 Da. Peptides with a probability score [-10Log_10_(*P*)] >70 were considered significant.” The detection of OspC, OspA, and GroEL are now mentioned in the main text, lines 116-118:**

**“Of note, the highest abundance proteins detected were OspC, OspA, and GroEL which are abundant and common contaminants in *B. burgdorferi* samples [46, 47].”**

2. Please provide an explanation for why the read coverage of the plasmids shown in Figure 5 is no greater than 29 kb even for plasmids larger than 29 kb.

**Thank you for the comment. As the reviewer points out, the average length for low frequency plasmids is <29kb (~8,000 bp on average) (Fig 5D). This may indicate a packaging bias towards shorter DNA molecules for non-cp32 DNA molecules. We now mention this on lines 161-64. Please also see our response to Reviewer #3, comment #2 on the maximum read lengths for each plasmid packaged.**

3. From the conserved motif analysis presented in Figure 8 the conservation of the pac site itself across species is unclear. How much sequence identity is needed for packaging? Is DNA from one species able to be packaged and transduced to another species?

**Thank you for mentioning this. How much sequence identity is needed for packaging and whether DNA from one species is able to be packaged and transduced to another species are excellent questions that we intend to answer in future studies. We have clarified that “significant matches to these motifs were not identified in other CA-11.2A genetic elements packaged by ϕBB-1 (Supplementary Data file 1), suggesting that packaging of non-cp32 DNA may require a pseudo-*pac* site that is smaller than the motifs identified or that packaging of non-cp32 DNA occurs spontaneously or through different mechanisms” on lines 243-247.**

4. Figures 8 and 9 are quite difficult to read. In particular, nucleic acid sequences in Figure 9 are unable to read due to the lack of contrast with the blue and green blocks.

**A higher resolution image of Figure 8 has been included in the revised manuscript. The nucleotides in Figure 9 have been enlarged and the background colors changed to provide greater contrast. The updated figures are shown below.**

**Figure 9. Full-length lp54 with fully resolved telomeres are recovered from ϕBB-1-packaged DNA. (A)** *De novo* assembly of packaged reads produced a 67,405-bp contig with tail-to-tail and head-to-head junctions. **(B and C)** Sequences at the packaged 5′ junction (light orange) or the 3′ junction (cyan) are compared to the lp54 reference sequence NC_012194.1. **(D)** Alignments of the tail-to-tail and head-to-head junctions reveals a variable 18-bp sequence in between the conserved inverted repeats. **(E and F)** Predicted hairpin structures are shown for each end of lp54. The loop sequence for each hairpin is underlined in panel D.

5. Some of the conclusions stated in the Discussion are too strongly worded. Line 347-348—suggests a possible role for, Lines 364-366—also suggests the possibility.

**We have revised the Discussion to temper the conclusions.**

Reviewer #3:

1. The studies were all carried out with strain CA-11.2A, the strain from which φBB-1 was initially isolated. Bacteriophage have been rarely observed with other Bb strains, although they all harbor the cp32 prophage. Further, CA-11.2A produces 20-100 fold more phage than B31 or 297 (the most commonly used Bb laboratory strains). The reason for this remains a mystery. It may be noteworthy that CA-11.2 is an ospC type D strain, a genotype not commonly observed in tick and human isolate studies. Is it possible that this lineage contains some unique sequences that may explain the behavior of CA-11.2A? Perhaps the authors could address this in the Discussion.

**Thank you for bringing up these observations. One possible explanation for why CA-11.2A produces more phage than B31 could be due to mutations in the *pac* region or other regulatory elements that govern phage replication decisions. Indeed, comparing the *pac* region in the B31 cp32s to the CA-11.2A cp32s reveal numerous differences upstream of the cut site we identified (Fig S3A). Notably the cut site itself is conserved across cp32s in B31 and CA-11.2A.**

**Phylogenetic analysis of the cp32 *pac* region in CA-11.2A and B31 reveal further insight. We find that the CA-11.2A cp32-1 isoform is packaged at a ~100-fold lower frequency than the other cp32 isoforms (see Fig 5B,C). Phylogenetic analysis reveals that the cp32-1 *pac* region is divergent from the other cp32 *pac* sites (Fig S3B). Similarly, prior observations in B31 by Wachter *et al.* (PMID: 36639656) find that the cp32-9 isoform is not transcriptionally active compared to the other cp32 isoforms. Our phylogenetic analysis reveals that the *pac* region of cp32-9 is also divergent (Fig S3B). These results suggest that mutations in the *pac* region can affect DNA packaging into phage capsids. We now discuss this on lines 219-233. We address the reviewer’s comments on phage production by different *B. burgdorferi* OspC types in point 3 below.**

**Figure S3. *B. burgdorferi* CA-11.2A and B31 *pac* region comparison. (A)** The pac region from the cp32s in *B. burgdorferi* B31 and CA-11.2A were aligned by ClustalW. The cut site is indicated by yellow arrows. **(B)** Phylogenetic analysis of the *pac* regions was performed by constructing an uncorrected neighbor joining tree.

2. P. 6 and fig. S1 – The data clearly demonstrate that reads covering the entire lengths of numerous plasmids other than cp32. The authors state that this suggests that full-length versions of these plasmids are packaged. Have the authors detected any reads (contigs) that would be large enough to represent full-length plasmids? It appears that non-cp32 plasmids do not have pac sequences so one would assume that these other DNA elements are packaged in the phage heads via some general transduction mechanism. Could the authors please comment.

**Thank you for the comment. The longest read for each plasmid are shown in the Table S2 below and in lines 154-55 in the revised manuscript. Interestingly, the longest reads for the cp32s were long enough to be dimers or even trimers in the case of cp32-5 and cp32-13. Whether these are biologically relevant is not clear. The maximum length reads for the other plasmids covered anywhere from ~46% of the reference sequence for lp54 to ~108% of the reference sequence for lp17.**

**Table S2. Maximum read lengths recovered for each element in the CA-11.2A genome.**

**Regarding the reviewer’s comment on plasmids not having *pac* sites, the mechanism by which these plasmids are packaged is not known. Our work however is consistent with prior work demonstrating that ϕBB-1 can package shuttle vectors that do not contain the *pac* region (shuttle vector pBSV2) and transfer them to other *B. burgdorferi* (PMID: 27811049).**

**One possibility explaining these observations is that the packaged DNA contains pseudo-*pac* sites, which facilitate packaging of non-phage DNA. Pseudo-*pac* site homology to bona fide *pac* sites affects generalized transduction frequencies by phages like P22 (PMID 776744, 6757664, 19066827). However, generalized transduction is unpredictable and any part of the bacterial genome is likely packaged at low frequencies (PMID: 35425613). We identified a 377-bp region that contains the ϕBB-1 *pac* site; however, the identity of the precise *pac* signal sequence (and any pseudo-*pac* signals in the *B. burgdorferi* genome) requires further investigation. We now discuss this on lines 342-351 of the revised manuscript.**

3. Fig. S1—Among the full-length plasmids packaged is lp56. This lp56 is quite different from the one found in B31, a portion of which comprises a full-length linearized cp32. The CA-11.2A lp56 is approximately 29 kb in length. The best BLAST matches for CA-11.2A lp56 are to lp56 from strains WI91-23, B331, B500 and B408. These strains all have a similar-sized lp56 and are all ospC type I. CA-11.2A lp56 encodes a PF32 sequence virtually identical to those of these other lp56 plasmids. With regard to the point raised in #1 above, might this strain “similarity” have any implication for the ability to produce greater amounts of phage? Please comment.

**Thank you for the comment. Outer surface lipoproteins can dramatically affect phage adsorption to bacterial cells (PMID: 32601452) and some phages encode their own lipoproteins that when expressed, protect the bacterial host from superinfection by other phages (PMID: 9812372). Different OspC types may differentially affect ϕBB-1 adsorption to *B. burgdorferi*, which may explain why *B. burgdorferi* strains that produce a lot of phage (e.g., CA-11.2A) have specific OspC types compared to strains that produce less phage (e.g., B31).**

4. P.10 – In a beautiful experiment, the authors demonstrate that attaching a cp32 pac site to pBSV2 results in packaging of the shuttle vector. Does this also proceed via a headful mechanism?

**Thank you for the compliment. Since general transducing phages overwhelmingly tend to be phages that package their genome by the headful mechanism, plasmids may also be packaged by a headful *pac* mechanism (please see our response to comment #1 regarding: pseudo-*pac* sites). More experimentation is required to determine if this is also true for ϕBB-1.**

5. In Table S1, the results of proteomic analysis of purified virions is presented. In addition to phage capsid proteins, the most abundant proteins are OspA and OspC. Is this simply because these proteins are so abundant in Bb or is there some other explanation?

**We suspect that the OspA and OspC lipoproteins are simply a contaminant that is residual from the *B. burgdorferi* culture. We now mention that OspC and OspA are the most abundant proteins identified in our proteomics analysis on lines 116-118. An interesting possibility is that lipoproteins such as OspC or OspA serve as phage receptors for ϕBB-1 that promote/block infection (please see our response to comment #3). If ϕBB-1 virions bind OspC and/or OspA, then this may help explain why they are so abundant.**

A few minor editing issues:
6. P.1, Abstract, ln 24 – “.. headful mechanism with preferentially packaging..”. This should read either preferential or delete “with” before “preferentially”.
7. P. 3, ln 94-95 – “re-chloroformed”. Is there such a word in the English language?
8. P. 17, ln 487 --- SM buffer is not defined.
9. p.18, ln509 – “sulfate” should be inserted after ammonium.

**Thank you for catching these. We have made the requested changes.**

10. The Bioproject has illumina reads as well as Nanopore reads. Not sure this matters, but they are not referred to anywhere in the manuscript.

**Thank you for noticing this. We used Illumina (MiSeq) technology to sequence the genome of our in-house CA-11.2A strain and these reads were included as part of the Bioproject. We now refer to the MiSeq Illumina reads on line 168.**
